# THE EXPERIENCES OF PEOPLE WITH COGNITIVE IMPAIRMENT DURING THE COVID-19 PANDEMIC IN ENGLAND

**DOI:** 10.1093/geroni/igac059.762

**Published:** 2022-12-20

**Authors:** Brian Beach, Paola Zaninotto

**Affiliations:** UCL (University College London), London, England, United Kingdom; UCL, London, England, United Kingdom

## Abstract

The COVID-19 pandemic presented challenges that may have impacted people with cognitive impairment in disproportionate ways. Using the ELSA COVID-19 sub-study collected in 2020, we examined the experiences of people across three cognitive function groups (no impairment, mild impairment, and dementia) with respect to a range of social and health outcomes, including: shielding and self-isolation; access to health and care services; changes in lifestyle behaviours during the pandemic, and the impacts on mental health, wellbeing, and other psychosocial measures. Differences among cognitive function groups varied according to both outcomes and time. For example, people with dementia were around 2.4 times more likely to be shielding in June/July than those with no impairment, but no difference was found for November/December. On many measures, people with dementia fared similarly to those with no impairment once controlling for other factors.

